# Revealing shared molecular and mechanistic signatures between intracranial aneurysms and abdominal aortic aneurysms: a comprehensive genomic analysis

**DOI:** 10.1186/s13023-025-03689-1

**Published:** 2025-04-24

**Authors:** Xiao Liu, Zhenjun Li, Hongzhen Xu, Wangqing He, Lei Wu, Bin Ji, Nuerzhati Nuermaimaiti, Guangnan Ao, Yuhang Feng, Xuying He

**Affiliations:** 1 Department of Neurosurgery, The Engineering Technology Research Center of Education Ministry of China, Guangdong Provincial Key Laboratory On Brain Function Repair and Regeneration, The Neurosurgery Institute of Guangdong Province, Zhujiang Hospital, Southern Medical University, The National Key Clinical Specialty, Guangzhou, 510282 China; 2 Brain Vascular Disease Center, Guangdong Second Provincial People’s Hospital, Guangzhou, 510317 China; 3 Department of Neurology, Taihe Hospital, Hubei University of Medicine, Shiyan, 442000 China; 4 Department of Neurosurgery, Renmin Hospital, Hubei University of Medicine, Shiyan, 442000 China

**Keywords:** Intracranial aneurysms, Abdominal aortic aneurysms, Immune-related, ITGA11

## Abstract

Intracranial aneurysms (IAs) and abdominal aortic aneurysms (AAAs) are both vascular diseases that are closely linked. However, the pathogenesis underlying the co-occurrence of IAs and AAAs remains poorly understood. This study aims to identify key biomarkers that shed light on the molecular mechanisms connecting these two diseases using bioinformatics analysis. Gene expression profiles (GSE122897, GSE237229) were obtained from the Gene Expression Omnibus (GEO) database. Differentially expressed genes (DEGs) common to both IAs and AAAs were identified and subjected to functional enrichment analysis. The Cytoscape cytoHubba plugin was used to identify hub genes, and their predictive ability was evaluated using the receiver operating characteristic (ROC) curve. Additionally, immune infiltration analyses and single-gene gene set enrichment analysis (GSEA) were conducted for the hub genes. A total of 46 DEGs were identified, including 40 upregulated genes and 6 downregulated genes. The common DEGs were found to be involved in extracellular matrix structural constituents, collagen fibril organization, and regulation of basic cellular processes. ITGA11 was identified as a key gene implicated in the comorbidity of IAs and AAAs, with its upregulation strongly associated with plasma cells. Furthermore, in both IAs and AAAs, glycosaminoglycan biosynthesis of extracellular matrix components and immune-related diseases were significantly linked to the high expression of ITGA11. Our findings suggest that the comorbidity of IAs and AAAs may be driven by shared inflammatory and immune response mechanisms, with ITGA11 emerging as a potential biomarker for this co-occurrence.

## Introduction

An aneurysm is a cerebral hemangiomatous protrusion resulting from abnormal changes in local blood vessels. In recent years, as living standards have improved and lifestyles and dietary habits have transformed, the prevalence of intracranial aneurysms (IAs) has been steadily increasing, with a noticeable shift toward a younger demographic affected by this condition. The formation of intracranial aneurysms is a complex, gradual process involving multiple pathways, such as cell apoptosis and inflammation. [1, 2]. Additionally, factors like hemodynamic shear stress, blood flow velocity [3], and estrogen deficiency have been shown to promote the development of IAs[4]. Ruptured intracranial aneurysms can lead to subarachnoid hemorrhage (SAH), which may result in death or disability [5]. The etiology of abdominal aortic aneurysms (AAAs) remains unclear. However, smoking, hypertension, chronic obstructive pulmonary disease (COPD), and peripheral vascular diseases are recognized as risk factors [6]. Most AAAs are degenerative and may be associated with atherosclerosis [7]. Infections, such as syphilis and fungal infections, as well as autoimmune conditions like Takayasu arteritis and giant cell arteritis, have also been linked to the occurrence of AAAs [8]. An AAA typically arises from congenital structural abnormalities or acquired pathological changes in the arterial wall, leading to localized weakness and reduced tension in the vascular wall. The continuous impact of blood flow causes permanent abnormal expansion or bulging, resulting in changes to the structure and tension of the vessel wall. Factors such as the degradation of the extracellular matrix (ECM), the generation of reactive oxygen species (ROS), oxidative stress injury, apoptosis, and dysfunction of vascular smooth muscle cells (VSMCs) all contribute to this process [9]. Ruptured AAAs can lead to massive abdominal bleeding or even death [10]. Several studies have suggested a higher incidence of IAs in patients with AAAs, and conversely, an elevated prevalence of AAAs in patients with IAs [11–13]. The shared pathogenic mechanisms between the two conditions appear to be linked to genetic mutations associated with hereditary diseases [14, 15]. Investigating the molecular mechanisms underlying IAs and AAAs, along with improving early detection and treatment of these diseases, holds significant clinical importance.

## Materials and methods

### Data finding and processing

**Fig. 1:**
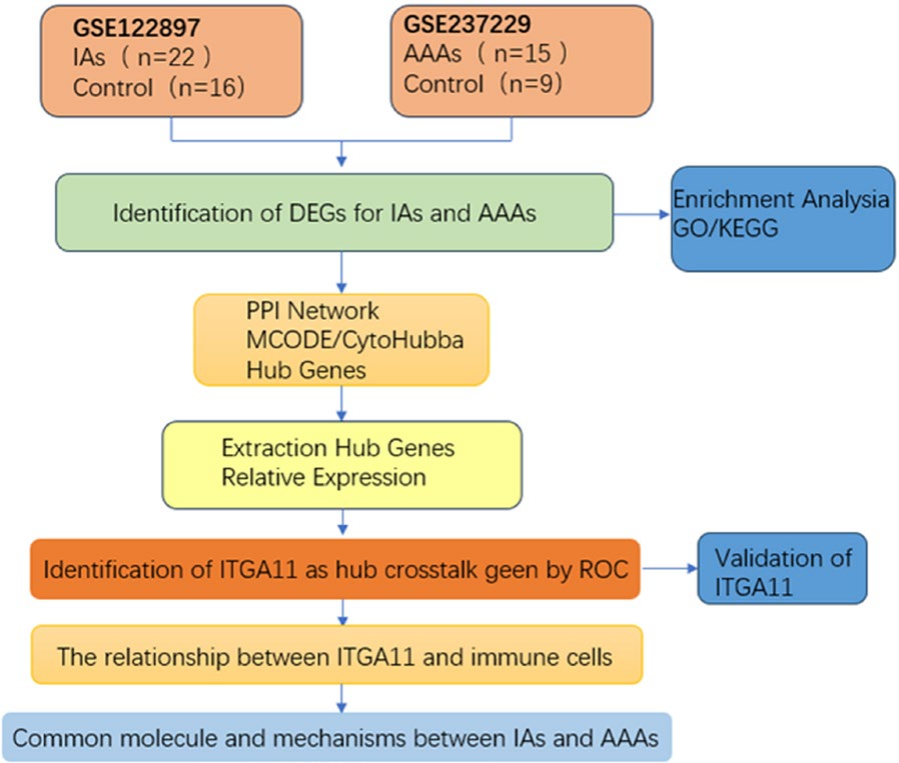
Analysis Process Flow

The workflow of this study is illustrated in (Fig. 1). We searched the keywords “intracranial aneurysm” and “abdominal aortic aneurysm” in the Gene Expression Omnibus (GEO) database. Datasets that included both aneurysm and normal tissue were selected, while datasets with small sample sizes, or data from peripheral blood or cerebrospinal fluid, were excluded. After removing duplicate subsets, the raw chip data for GSE122897 and GSE237229 were downloaded for analysis. GSE122897 contained vascular tissue data from 22 patients with intracranial aneurysms and 16 control subjects. GSE237229 included tissue data from 15 patients with abdominal aortic aneurysms and 9 control subjects. The validation dataset, GSE75436, comprised vascular tissues from 15 patients with intracranial aneurysms and 15 controls. GSE57691 contained tissue data from 49 patients with abdominal aortic aneurysms and 10 control subjects. More detailed information about the included datasets is provided in (Table 1).

**Table 1:** Information regarding the incorporated datasets

Dataset	Platform	Samples	Disease	Group
GSE122897	GPL16791	22 patients and 16 controls	IA	Discovery
GSE237229	GPL24676	15 patients and 9 controls	AAA	Discovery
GSE75436	GPL570	15 patients and 15 controls	IA	Validation
GSE57691	GPL10558	49 patients and 10 controls	AAA	Validation

### Differentially expressed gene screening

Differentially expressed genes (DEGs) between the disease and control groups were identified using the"limma"package in R software. The screening criteria applied were logFC (fold change) > 2 and *p*-value < 0.05. Common DEGs shared by intracranial aneurysms (IAs) and abdominal aortic aneurysms (AAAs) were identified using the"VennDiagram"package. These common DEGs were subsequently retained for further analyses.

### Functional enrichment analyses of the DEGs

To further explore the underlying biological processes, we analyzed the composition, molecular functions, and pathways of overlapping DEGs. Gene Ontology (GO) analysis and Kyoto Encyclopedia of Genes and Genomes (KEGG) pathway enrichment analysis were performed using the online tool Database for Annotation, Visualization and Integrated Discovery (DAVID) (https://david.ncifcrf.gov/). This analysis aimed to transform raw data into meaningful biological insights. The results were visualized using the"ggplot2"package.

### Construct the protein–protein interaction network and recognition hub gene

To gain deeper insights into the interactions among DEGs, the STRING network analysis tool (https://string-db.org/) was used to construct protein–protein interaction (PPI) networks, revealing their molecular mechanisms. The resulting PPI network was visualized using Cytoscape software. Additionally, the Molecular Complex Detection (MCODE) plugin was applied to identify highly interconnected gene clusters within the PPI network. The selection criteria in MCODE were as follows: MCODE score ≥ 4, degree cutoff = 2, node score cutoff = 0.2, k-core = 2, and max depth = 100. Furthermore, the Cytoscape plugin CytoHubba was utilized to identify hub crosstalk genes.

### Confirm the correlation between the hub gene and immune infiltrating cells

Spearman’s correlation analysis was performed in R software to evaluate the relationship between the expression levels of hub genes and the proportions of infiltrating immune cells.

### ROC analysis

To further evaluate the hub crosstalk gene as a common potential indicator for IAs and AAAs, receiver operating characteristic (ROC) analyses were performed using the datasets GSE122897 and GSE237229, with GSE75436 and GSE57691 serving as validation datasets. The area under the ROC curve (AUC) was used to assess diagnostic accuracy. The R package"pROC"was employed for data analysis and visualization. An ideal AUC value is 1, indicating perfect diagnostic ability, while values greater than 0.5 suggest predictive capability. Higher AUC values reflect greater specificity and sensitivity.

### Verify the genes that are most specific and sensitive

Following ROC analysis, the gene with the largest area under the curve (AUC) was identified as the most specific and sensitive marker. Expression values for this gene were then extracted from the corresponding datasets, GSE122897 and GSE237229, for further analysis. Validation was subsequently performed using the datasets GSE75436 and GSE57691.

### Associations between the most specific and sensitive genes and the immune cell landscape

The gene file for 22 types of immune cells was downloaded from the CIBERSORT website (http://CIBERSORT.stanford.edu/). Statistical tests were performed using R software with one thousand permutations. The results provided the percentage ratios of immune cell types for all samples, ensuring that the sum of immune cell ratios for each sample equaled 1. Data with *p*-values < 0.05 were filtered from the CIBERSORT results for further analysis. The filtered data were then integrated to generate a comprehensive matrix of immune cell proportions. The visualization of CIBERSORT results was conducted using the R package"vioplot."Additionally, the most specific and sensitive genes identified in the previous ROC analysis were selected for correlation analysis to investigate their relationships with immune cells.

## Results

**Fig. 2:**
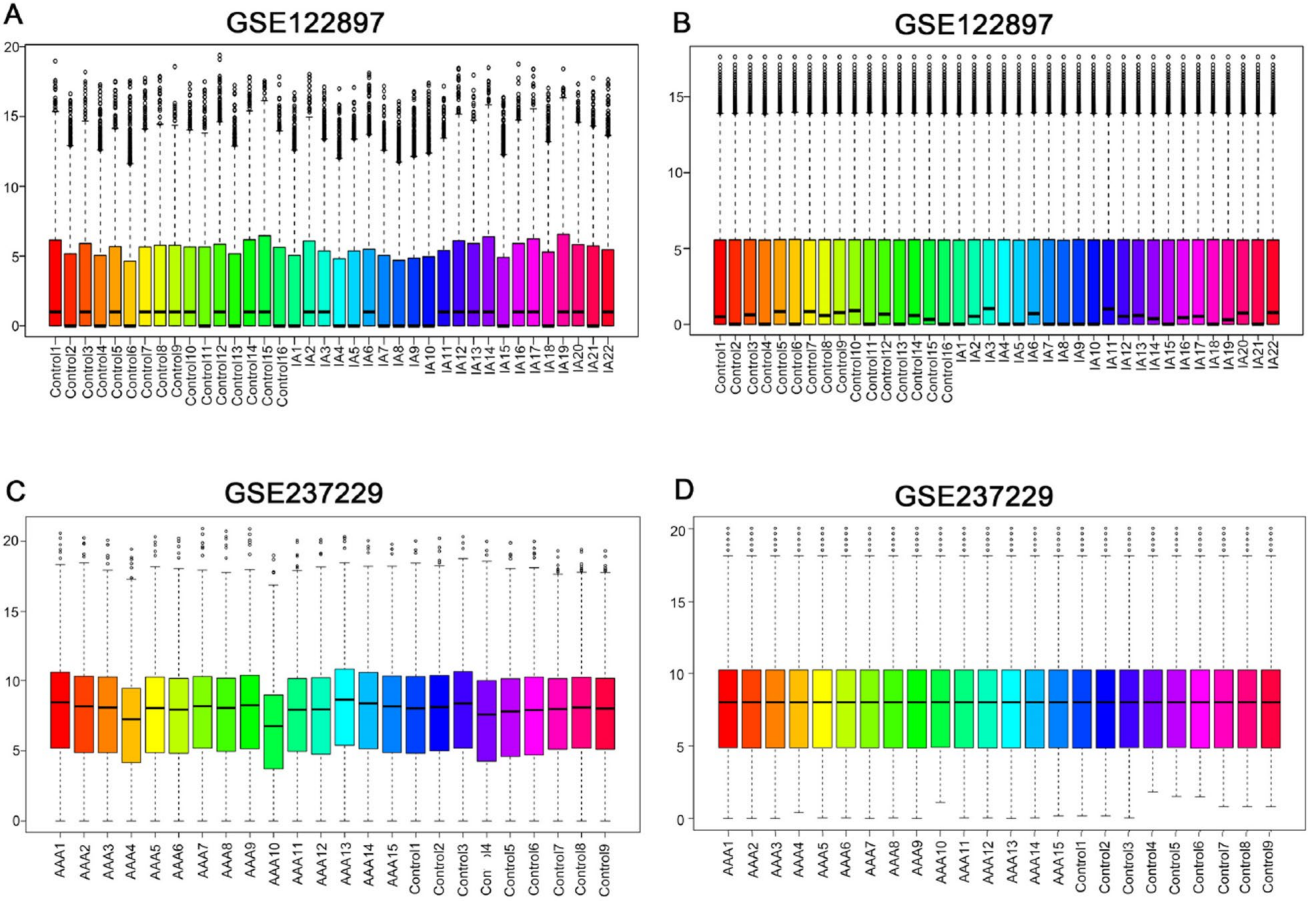
Box plots of sample expression before normalization (left) and after normalization (right). The x-axis represents sample information, and the y-axis represents gene expression values. **A** Before normalization of IA and control groups. **B** After normalization of IAs and control groups. **C** Before normalization of AAAs and control groups. **D** After normalization of AAAs and control groups

**Fig. 3:**
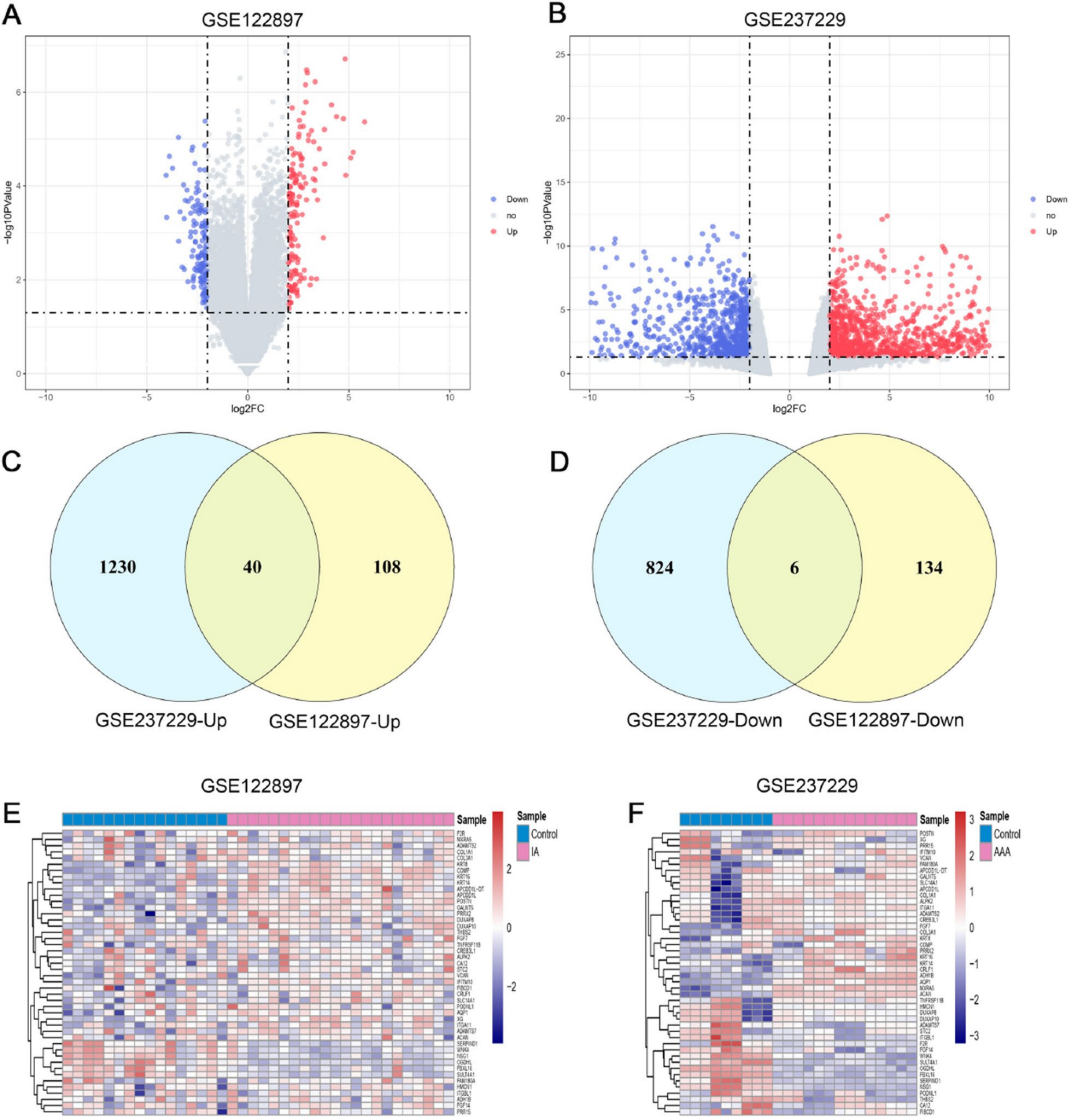
**A** A volcano plot displays data from all genes, with each dot representing one gene. Genes meeting the screening criteria are depicted in blue (for downregulated) and red (for up-regulated), while the remaining genes are shown in gray. DEGs are shown on a volcano plot in GSE122897. **B** DEGs are shown on a volcano plot in GSE237229. **C** Venn diagram of upregulated DEGs shared by IAs and AAAs. **D** Venn diagram of downregulated DEGs shared by IAs and AAAs. **E** The top 46 DEGs shown in GSE122897 are displayed on a heatmap. **F** The top 46 DEGs shown in GSE237229 are displayed on a heatmap. DEGs, differentially expressed genes; IAs, Intracranial Aneurysm; AAAs, Abdominal Aortic Aneurysm

After normalizing the expression matrix (Fig. 2), differential expression analysis was performed. A total of 288 genes were identified between the IAs group and the control group, including 148 up-regulated genes and 140 down-regulated genes. For the AAAs group, 2100 genes were identified in comparison to the control group, including 1270 up-regulated genes and 830 down-regulated genes. The results of the gene expression analysis are shown in the volcano plots in Fig. 3A, B.Next, we identified the intersection of the two diseases, which included 40 up-regulated genes and 6 down-regulated genes (Fig. 3C, D). The common differential genes are presented in the heatmap (Fig. 3E, F). Interestingly, ITGA11 was highly expressed in both IAs and AAAs compared to the control group.

### Functional and pathway enrichment

**Fig. 4:**
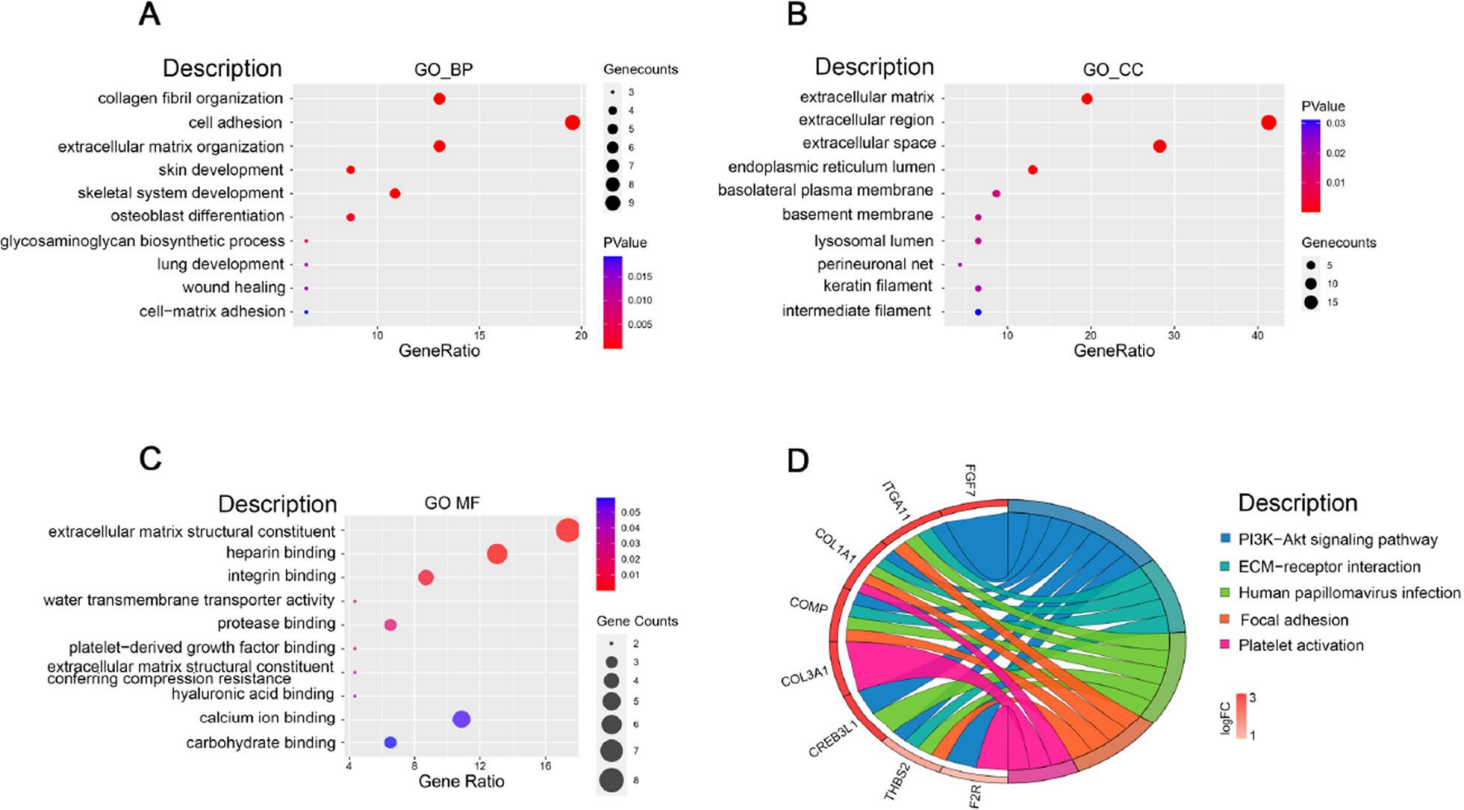
**A** Gene Ontology (GO) enrichment analysis of Biological Processes (BP) **B** Gene Ontology (GO) enrichment analysis of Cellular Components (CC) **C** Gene Ontology (GO) enrichment analysis of Molecular Functions (MF) **D** Kyoto Encyclopedia of Genes and Genomes (KEGG) enrichment analysis results

The results of the Reactome gene set enrichment analysis for the co-expressed differential genes of IAs and AAAs are shown in Fig. 4. These co-expressed genes were primarily involved in biological processes such as collagen fiber formation, cell adhesion, and extracellular matrix organization (Fig. 4A). Interestingly, the cellular components and molecular functions of these genes were predominantly associated with the extracellular matrix and its components (Fig. 4B, C).

The KEGG pathway enrichment analysis of the co-expressed differential genes revealed significant involvement in the PI3 K-Akt signaling pathway, ECM-receptor interaction, and human papillomavirus infection, among other pathways (Fig. 4D). Both the GO and KEGG enrichment results highlighted a strong association between DEGs and the extracellular matrix (ECM).

### Identification of the hub gene was achieved through the PPI network and modular screening technique

**Fig. 5:**
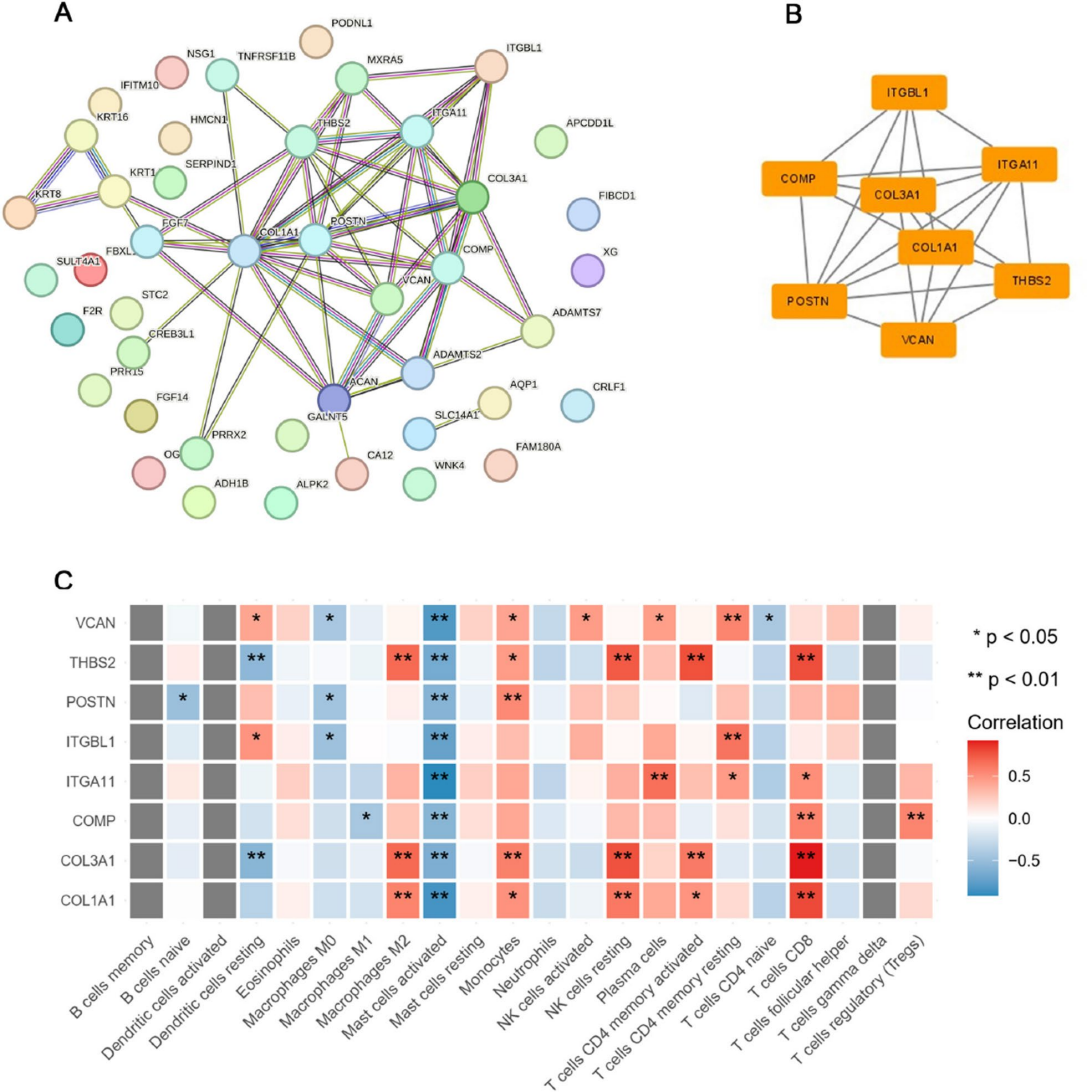
**A** String-based PPI network with shared DEGs **B** Utilizing Cytoscape’s MCODE plug-in, the cluster subnet brings to light hub genes within the PPI network. Nodes and edges visually depict cluster members and their interconnections **C** The heatmap illustrates the correlation between central genes and the ratio of immune cells. Hub gene expression is depicted on the y-axis, while immune cells are represented on the x-axis. The positive correlation is indicated by red, the negative correlation by blue, and the intensity of color saturation reflects the strength of the correlation, as depicted in the accompanying barchart on the right

**Fig. 6:**
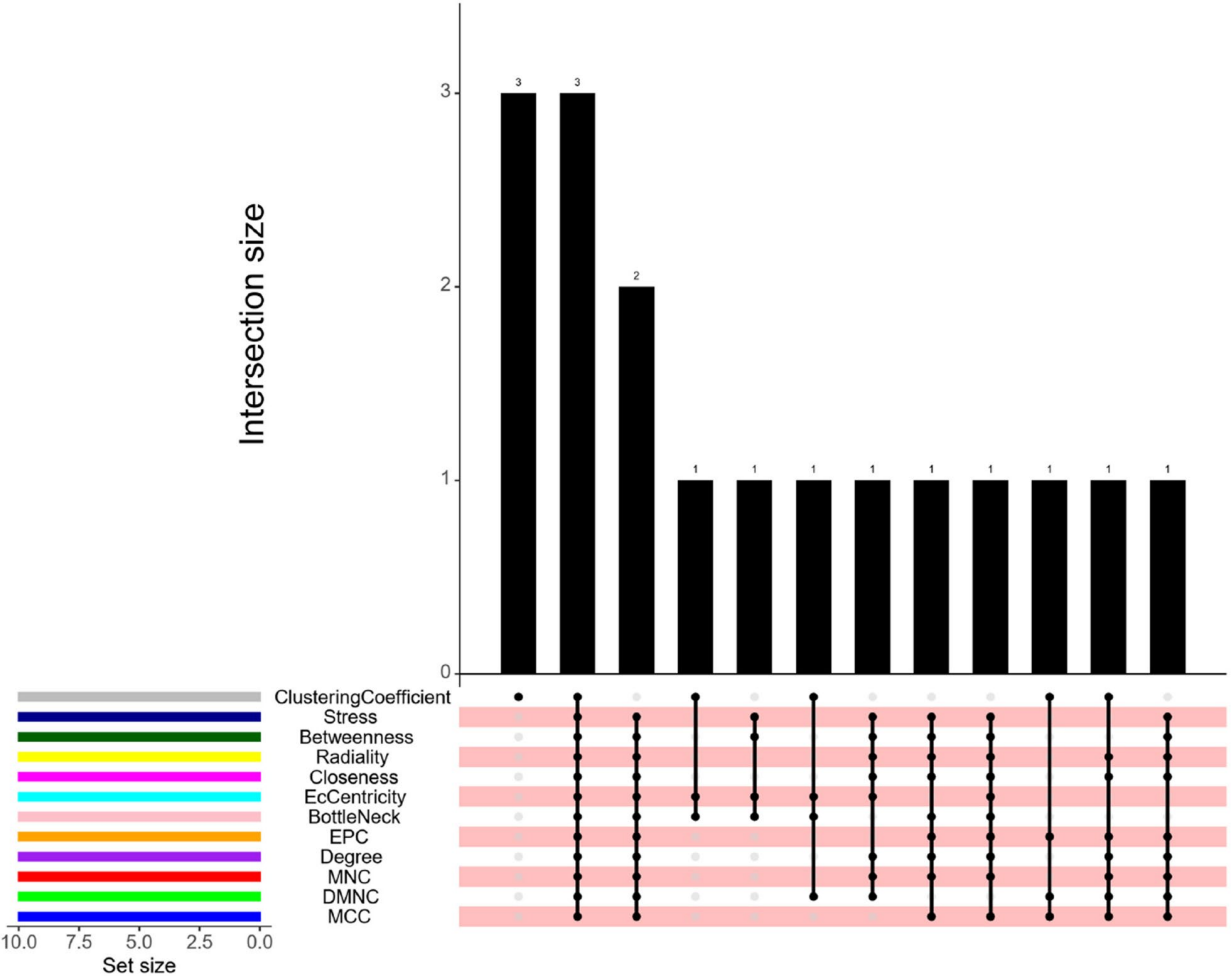
Hub crosstalk genes are obtained through twelve algorithms provided by the CytoHubba Cytoscape plugin, designed for identifying hub nodes within the network. The intersections of sets are visualized as a matrix using the R package”UpSet.” Different colors in the legend, located in the bottom left, represent distinct algorithms. The y-axis of the bar plot indicates the number of intersecting genes, while the x-axis displays the set names. The matrix depicts which sets intersect with each other

To identify the hub genes, we conducted a PPI network analysis of the 46 DEGs using the STRING database (Fig. 5A). The resulting network diagram, shown in Fig. 5B, includes 56 edges and 43 nodes. The most significant cluster of hub genes consisted of ITGA11, VCAN, THBS2, POSTN, ITGBL1, COMP, COL1 A1, and COL1 A3. Subsequent correlation analysis revealed a positive correlation between these hub genes and various immune infiltrating cells, including M2 macrophages, monocytes, resting NK cells, CD8 + T cells, and plasma cells, among 22 other types of immune cells. Conversely, a negative correlation was observed with activated mast cells (Fig. 5C). We then used the CytoHubba plugin in Cytoscape to identify the central genes. All 12 algorithms pointed to ITGA11, VCAN, and THBS2 as the central crosstalk genes between IAs and AAAs (Fig. 6). Table 2 lists the top 10 hub genes ranked by the CytoHubba plugin.

**Table 2:** The CytoHubba plugin ranks the top 10 hub genes

Rank	**MCC**	**DMNC**	MNC	Degree	EPC	BottleNeck	Eccentricity	closeness	Radiality	Betweenness	Stress	Clusteringcoefficient
1	C0L1 A1	ITGA11	C0L1 A1	C0L1 A1	COL1 A1	COL1 A1	FGF7	COL1 A1	COL1 A1	COL1 A1	COL1 A1	TNFRSF11B
2	POSTN	ITGBL1	POSTN	POSTN	POSTN	POSTN	COL1 A1	POSTN	POSTN	KRT14	ACAN	PRRX2
3	COL3 A1	MXRA5	COL3 A1	COL3 A1	COL3 A1	COL3 A1	TNFRSF11B	COL3 A1	COL3 A1	ACAN	KRT14	KRT8
4	ITGA11	VCAN	COMP	COMP	COMP	KRT14	ADAMTS2	COMP	COMP	POSTN	POSTN	KRT16
5	THBS2	THBS2	ACAN	ACAN	ITGA11	ACAN	THBS2	ACAN	ACAN	COMP	COMP	MXRA5
6	ITGBL1	COL3 A1	THBS2	THBS2	THBS2	TNFRSF11B	VCAN	THBS2	FGF7	COL3 A1	COL3 A1	ITGBL1
7	COMP	POSTN	ITGA11	ITGA11	VCAN	ADAMTS2	ITGA11	ITGA11	THBS2	FGF7	FGF7	ADAMTS2
8	VCAN	ADAMTS2	VCAN	VCAN	ITGBL1	THBS2	POSTN	VCVN	VCAN	THBS2	THBS2	ITGA11
9	MXRA5	COMP	ITGBL1	ITGBL1	ACAN	VCAN	COL3 A1	FGF7	ITGA11	VCAN	VCAN	VCAN
10	ACAN	FGF7	FGF7	FGF7	MXRA5	ITGA11	KRT14	ITGBL1	ITGBL1	ITGA11	ITGA11	THBS2

### Diagnostic effect evaluation

**Fig. 7:**
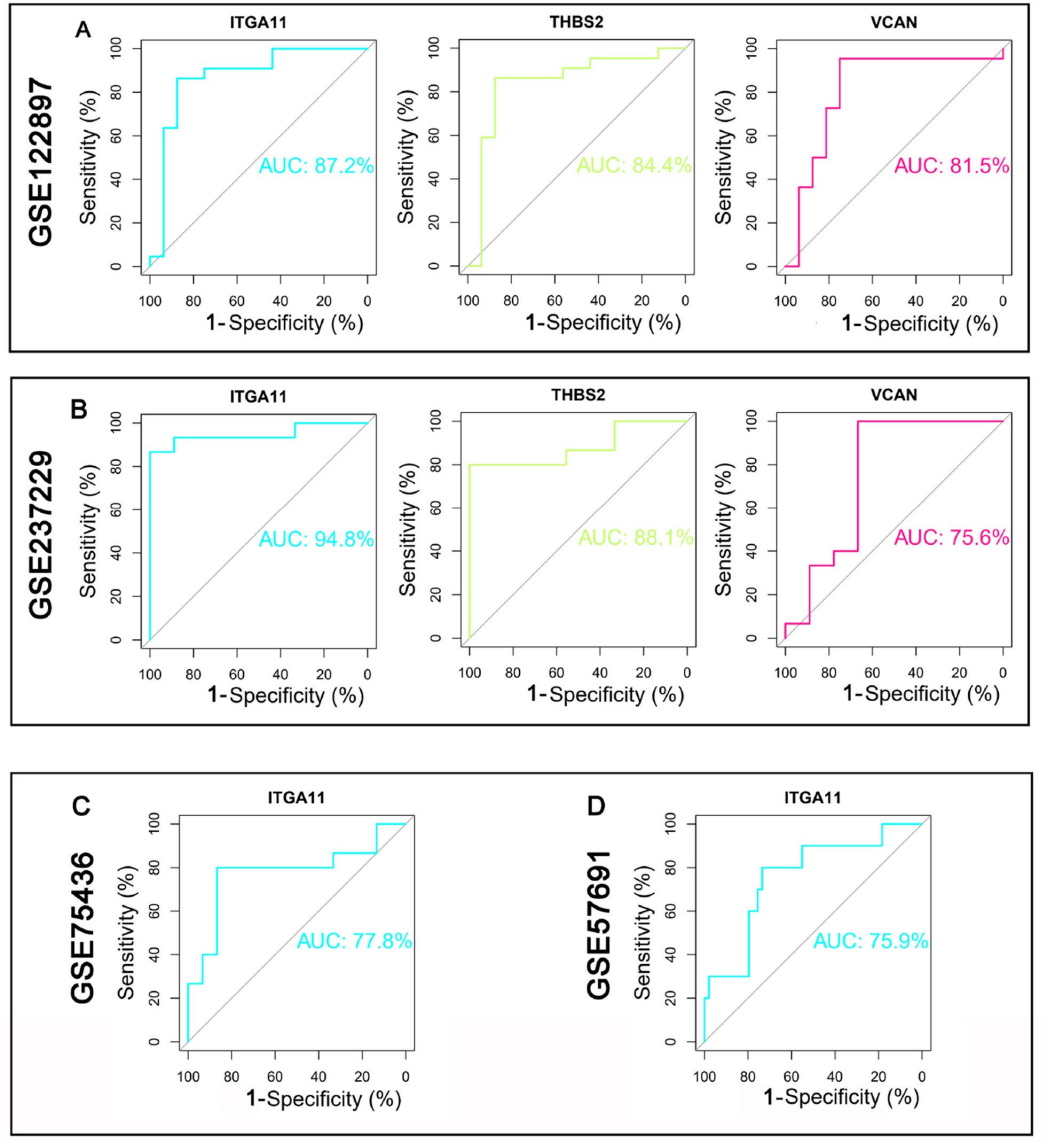
Using receiver operating characteristic (ROC) analysis to examine candidate hub genes. The x-axis represents specificity, the y-axis represents sensitivity, and AUC represents the area under the curve. **A** ROC curves of three candidate genes (ITGA11, THBS2, VCAN) in GSE122897. **B** ROC curves of three candidate genes (ITGA11, THBS2, VCAN) in GSE237229. **C** Validation of the most significant diagnostic gene, ITGA11, in datasets GSE75436 and GSE57691

To evaluate the potential of the selected DEGs as co-expression biomarkers for both IAs and AAAs, ROC analyses were performed on these genes. The results of the ROC curve analysis revealed that ITGA11 had the highest predictive power for both diseases, with AUC values of 0.872 and 0.948. The detailed results are shown in Fig. 7.

### Validation of ITGA11

**Fig. 8:**
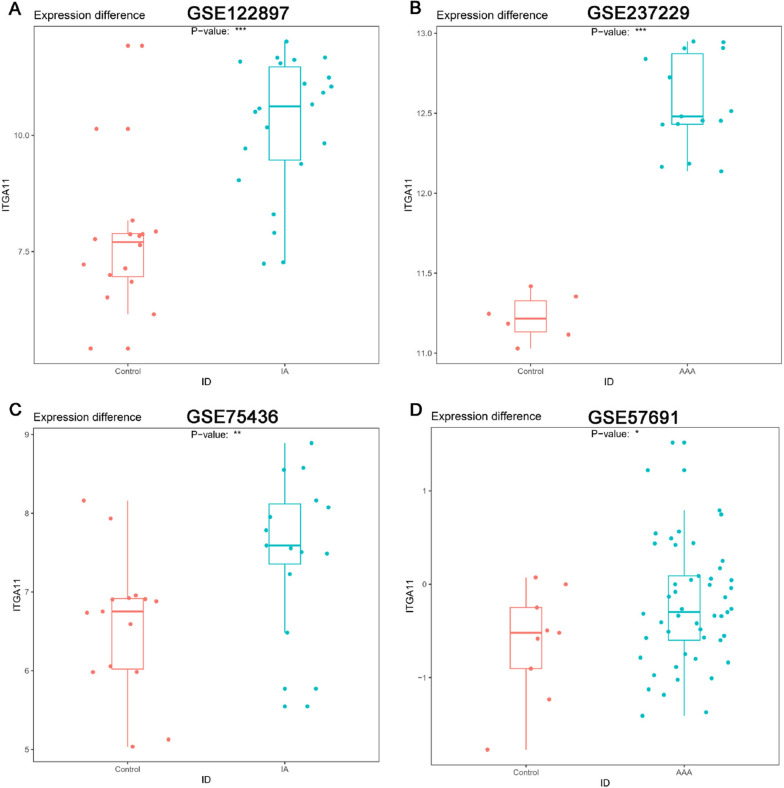
Expression and diagnostic significance of ITGA11 in IAs and AAAs. **A** Expression levels of ITGA11 in IAs patients were determined using GSE122897. **B** Expression levels of ITGA11 in AAAs patients were determined using GSE237229. **C** Validation of ITGA11 expression levels in IAs patients using GSE75436. **D** Validation of ITGA11 expression levels in AAAs patients using GSE57691. IAs, Intracranial Aneurysm; AAAs, Abdominal Aortic Aneurysm. *** *p* value < 0.001, **p value < 0.01, *p value < 0.05

We extracted the expression values of ITGA11 in IAs and AAAs cases and found that they were higher than those in the control group (Fig. 8A, B). For validation, GSE75436 was used for IAs, and GSE57691 was used for AAAs. In both cases, ITGA11 was up-regulated compared to the control group (Fig. 8C, D).

### Relationship between ITGA11 and immune cell landscape

**Fig. 9:**
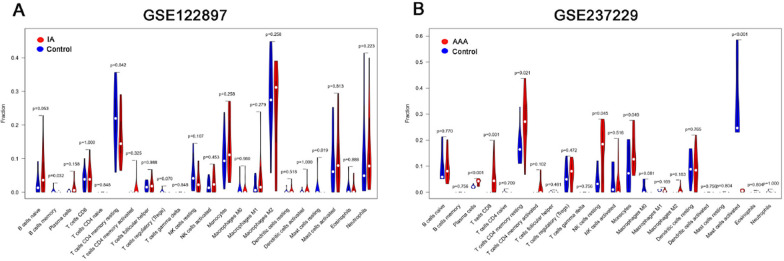
The immune infiltration landscape between disease and control groups. Violin plots of immune cell proportions. The y-axis represents the proportions of immune cells. **A** Immune infiltration status in IAs patients. **B** Immune infiltration status in AAAs patients

**Fig. 10:**
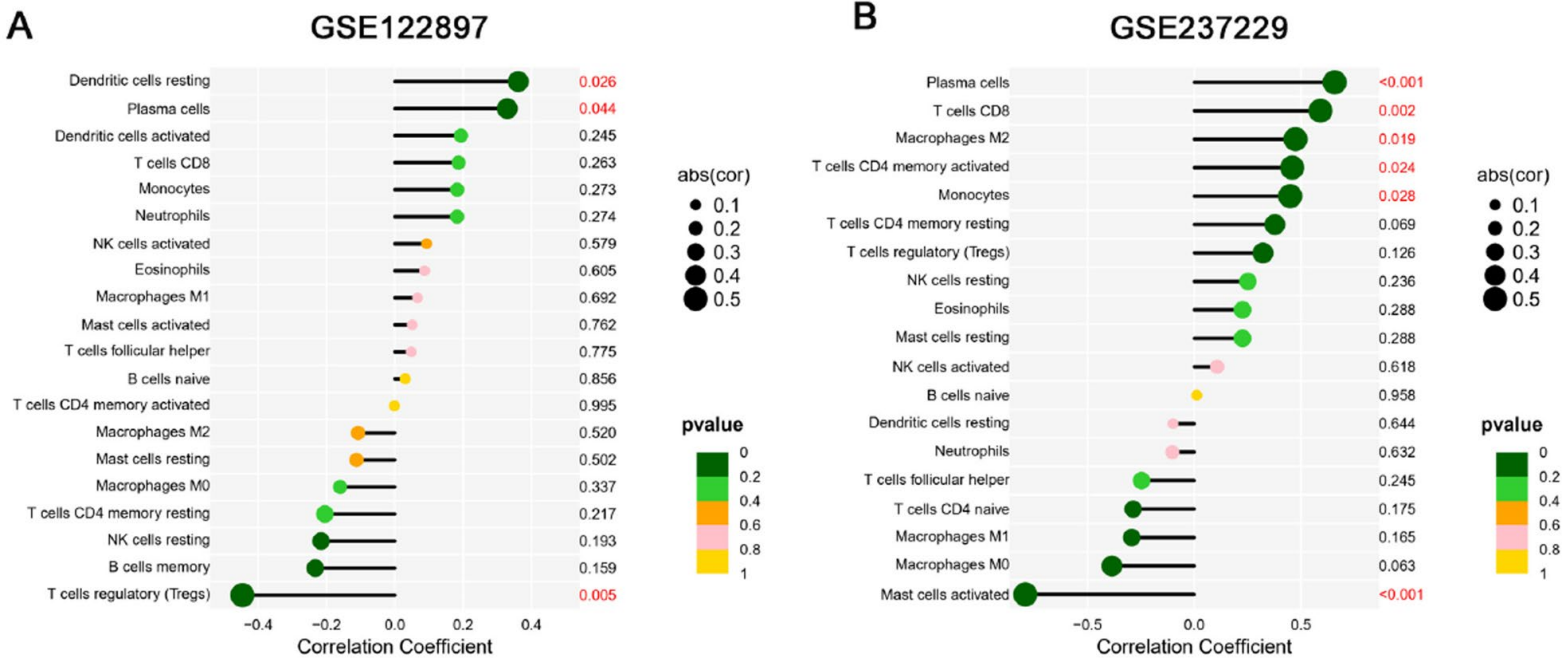
The lollipop chart illustrates the correlation between ITGA11 and immune infiltrating cells. The y-axis represents immune infiltrating cells, with a positive correlation on the right side of the axis and a negative correlation on the left side. The size of the spheres indicates the strength of correlation, while the p-value is shown in the bar chart on the right. **A** Relationship between ITGA11 and Immune Infiltrating Cells in IAs. **B** Relationship between ITGA11 and immune infiltrating cells in AAAs

According to the results of the CIBERSORT algorithm, the immune status of patients with IAs and AAAs was significantly altered compared to the control group (Fig. 9). Additionally, correlation analysis revealed a significant association between ITGA11 and plasma cells in both diseases (Fig. 10).

### GSEA analysis of ITGA11

**Fig. 11:**
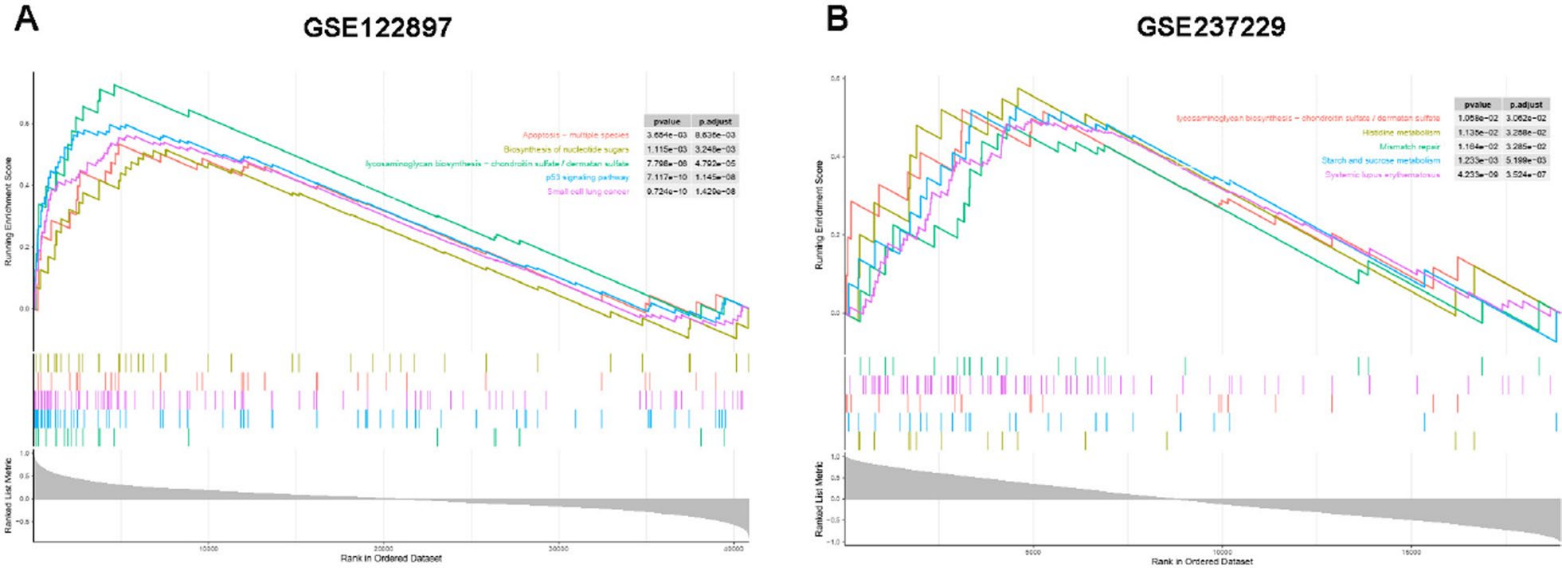
The upper part of the figure illustrates the increasing and decreasing trend of enrichment scores (ES) for predefined gene sets across different enriched pathways, distinguished by colors. The middle part of the figure represents the position of members of predefined gene sets within enriched pathways. The bottom part of the figure displays genes sorted from high to low according to a certain metric. **A** Single-gene GSEA of ITGA11 in IAs using GSE122897. **B** Single-gene GSEA of ITGA11 in AAAs using GSE237229. GSEA, Gene Set Enrichment Analysis

GSEA enrichment analysis of the single gene ITGA11 in IAs revealed the following pathways: multispecies apoptosis, biosynthesis of nucleotide sugars, glycosaminoglycan biosynthesis (chondroitin sulfate/dermatan sulfate), p53 signaling pathway, and small cell lung cancer (Fig. 11A). In AAAs, the enriched pathways included glycosaminoglycan biosynthesis (chondroitin sulfate/dermatan sulfate), histidine metabolism, mismatch repair, starch and sucrose metabolism, and systemic lupus erythematosus (Fig. 11B). Interestingly, most of the pathways identified in both IAs and AAAs are related to immunoinflammatory and the extracellular matrix (ECM).

## Discussion

An aneurysm is characterized by a permanent and irreversible local dilation of an artery. The global prevalence of intracranial aneurysms (IAs) is estimated to be between 2 and 5% [16–18], while abdominal aortic aneurysms (AAAs) affect approximately 5.5% of the population [19]. Aneurysmal subarachnoid hemorrhage, which occurs after the rupture of an IA, accounts for 5% of all strokes. Compared to other types of stroke, it has a poor prognosis and is associated with high rates of morbidity and mortality [20]. Similarly, the rupture of an AAA can result in life-threatening intra-abdominal bleeding, with mortality rates ranging from 65 to 85% [21]. The correlation between intracranial aneurysms (IAs) and abdominal aortic aneurysms (AAAs) is becoming increasingly apparent. However, few studies have explored their shared pathogenesis and common biomarkers. In this study, we identified 46 genes shared by IAs and AAAs, which are enriched in biological functions and pathways primarily associated with immunity, inflammation, and the extracellular matrix (ECM). The extracellular matrix plays a critical role in immunity and inflammation, as the process of blood vessel remodeling induced by inflammation is largely mediated by ECM proteins and proteases. Matrix remodeling and vascular regression are essential for resolving inflammatory responses and promoting tissue repair [22]. Further analysis revealed that ITGA11 is a central crosslinker gene in the comorbidity of IAs and AAAs. Additionally, ITGA11 may regulate this comorbidity through immunoinflammatory pathways.

Some studies suggest that aneurysm formation is caused by increased hemodynamic stress, which destabilizes the vascular wall, leading to morphological changes and potential rupture. The primary cellular mechanism for adapting to heightened hemodynamic stress involves the proliferation and migration of smooth muscle cells (SMCs) into the vessel cavity [23]. This process triggers significant inflammatory and proliferative responses in the surrounding tissues [24]. In IAs, endothelial dysfunction appears to be the main driver, involving an inflammatory response from macrophages and SMCs, ultimately leading to the apoptotic degradation of the extracellular matrix (ECM) and SMCs [25]. Inflammation is thus a critical factor in the pathogenesis of IAs [26]. Similarly, in AAAs, key pathological features include ECM remodeling, vascular smooth muscle degeneration, cell loss, and the accumulation and activation of inflammatory cells [27, 28]. Inflammation also plays a vital role in AAAs, significantly influencing factors related to aortic wall remodeling. Among these, monocytes and macrophage subpopulations play distinct and pivotal roles in initiating, developing, and healing aneurysms [29]. While ECM was once considered an inert framework, studies now highlight its dynamic role as a partner of the immune system [30].ECM not only provides structural support for the physiological activities of tissue cells but also plays a crucial role in immune regulation during both homeostasis and pathological states. Conversely, the immune system helps maintain stromal microenvironment homeostasis and repairs stromal integrity after injury [31, 32].

ITGA11 (integrin α11) was initially identified in differentiated fetal muscle cells and is predominantly expressed in fibroblasts and mesenchymal non-muscle cells [33]. As a major collagen receptor, it recognizes the GFOGER sequence in interstitial collagen, facilitating cell invasion and migration [34]. ITGA11 has been reported to enhance the tumorigenicity of lung cancer by modulating IGF-2 [35]. Additionally, it plays a critical role in promoting skin inflammation, as demonstrated in Aldara-induced psoriasiform dermatitis (AIPD) [36].

In contrast to other widely studied integrin members, ITGA11 has been rarely reported in studies related to aneurysm formation. Our study identified ITGA11 as a central crosstalk gene between IAs and AAAs. Enrichment analysis of the common DEGs revealed the involvement of inflammatory, immune, and extracellular matrix pathways in the comorbidities of IAs and AAAs. Immune infiltration analysis further demonstrated significant differences in immune status between patients with IAs and AAAs and the control group. Additionally, correlation analysis indicated a strong association between ITGA11 expression and Plasma cells. Single-gene-based GSEA identified pathways such as multispecies apoptosis, glycosaminoglycan biosynthesis (chondroitin sulfate/dermatan sulfate), the P53 signaling pathway, and immune-related disease interactions. These findings suggest that ITGA11 may regulate the comorbidities of IAs and AAAs through mechanisms involving immune and inflammatory regulation, extracellular matrix interactions, and cell biological processes. Thus, ITGA11 may serve as a potential biomarker for the early detection and prevention of complications associated with IAs and AAAs.

The study has several limitations. First, the sample sizes in the datasets GSE122897 and GSE237229 are relatively small, which may introduce some deviations in the analysis and calculations. Second, due to the low incidence of IAs and AAAs complications, we were unable to collect clinical samples and identify microarray data within the limited time frame. This limitation may lead to an overinterpretation of the data. Third, our findings have not been validated through subsequent experiments, and thus, they require in vitro experimental support as well as a larger number of follow-up clinical samples for actual clinical application.

## Conclusions

This study provides new insights into the shared pathogenesis of intracranial aneurysms (IAs) and abdominal aortic aneurysms (AAAs). Using bioinformatics analysis, we identified the composition of immune cell infiltration and highlighted the central role of ITGA11 in the comorbidity of IAs and AAAs. ITGA11 regulates key immunoinflammatory pathways, extracellular matrix interactions, and cellular biological processes, and may serve as a promising biomarker for the early detection and prevention of complications associated with IAs and AAAs. These findings pave the way for future studies to validate ITGA11 as a target for clinical application.

## Data Availability

The GEO dataset used in this study is publicly available at (https://www.ncbi.nlm.nih.gov/geo/).

## References

[1] Klopf J, Brostjan C, Neumayer C, Eilenberg W. Neutrophils as regulators and biomarkers of cardiovascular inflammation in the context of abdominal aortic aneurysms. Biomedicines. 2021;9(9):1236. 10.3390/biomedicines9091236. 34572424 10.3390/biomedicines9091236 PMC8467789

[2] Juvela S, Porras M, Heiskanen O. Natural history of unruptured intracranial aneurysms: a long-term follow-up study. J Neurosurg. 1993;79(2):174–82. 8331397 10.3171/jns.1993.79.2.0174

[3] Cebral JR, Duan X, Gade PS, Chung BJ, Mut F, Aziz K, Robertson AM. Regional mapping of flow and wall characteristics of intracranial aneurysms. Ann Biomed Eng. 2016;44(12):3553–67. 27350071 10.1007/s10439-016-1682-7 PMC5114167

[4] Jamous MA, Nagahiro S, Kitazato KT, Tamura T, Kuwayama K, Satoh K. Role of estrogen deficiency in the formation and progression of cerebral aneurysms. Part II: experimental study of the effects of hormone replacement therapy in rats. J Neurosurg. 2005;103(6):1052–7. 10.3171/jns.2005.103.6.1052. 16381192 10.3171/jns.2005.103.6.1052

[5] Neifert SN, Chapman EK, Martini ML, Shuman WH, Schupper AJ, Oermann EK, Mocco J, Macdonald RL. Aneurysmal subarachnoid hemorrhage: the last decade. Transl Stroke Res. 2021;12(3):428–46. 33078345 10.1007/s12975-020-00867-0

[6] Sörelius K, Budtz-Lilly J, Mani K, Wanhainen A. Systematic review of the management of mycotic aortic aneurysms. Eur J Vasc Endovasc Surg. 2019;58(3):426–35. 31320247 10.1016/j.ejvs.2019.05.004

[7] Nuche J, Palomino-Doza J, Ynsaurriaga FA, Delgado JF, Ibáñez B, Oliver E, Subías PE. Potential molecular pathways related to pulmonary artery aneurysm development: lessons to learn from the aorta. Int J Mol Sci. 2020;21(7):2509. 10.3390/ijms21072509. 32260370 10.3390/ijms21072509 PMC7177585

[8] Brownstein AJ, Kostiuk V, Ziganshin BA, Zafar MA, Kuivaniemi H, Body SC, Bale AE, Elefteriades JA (2018) Genes associated with thoracic aortic aneurysm and dissection: 2018 update and clinical implications. Aorta, **6**(1):13-20 10.1055/s-0038-1639612 PMC6136681 30079932

[9] Golledge J. Abdominal aortic aneurysm: update on pathogenesis and medical treatments. Nat Rev Cardiol. 2019;16(4):225–42. 30443031 10.1038/s41569-018-0114-9

[10] Sakalihasan N, Limet R, Defawe OD. Abdominal aortic aneurysm. Lancet. 2005;365(9470):1577–89. 15866312 10.1016/S0140-6736(05)66459-8

[11] Goyal MS, Gottumukkala R, Bhalla S, Kates A, Zipfel GJ, Derdeyn CP. Bicuspid aortic valves and thoracic aortic aneurysms in patients with intracranial aneurysms. Neurology. 2015;84(1):46–9. 25428688 10.1212/WNL.0000000000001104 PMC4336099

[12] Shin YW, Jung KH, Moon J, Lee ST, Lee SK, Chu K, Roh JK. Site-Specific relationship between intracranial aneurysm and aortic aneurysm. Stroke. 2015;46(7):1993–6. 25991415 10.1161/STROKEAHA.115.009254

[13] Lee D, Ahn SJ, Cho ES, Kim YB, Song SW, Jung WS, Suh SH. High prevalence of intracranial aneurysms in patients with aortic dissection or aneurysm: feasibility of extended aorta CT angiography with involvement of intracranial arteries. J Neurointerv Surg. 2017;9(10):1017–21. 27609114 10.1136/neurintsurg-2016-012619

[14] Kim ST, Brinjikji W, Lanzino G, Kallmes DF. Neurovascular manifestations of connective-tissue diseases: a review. Interv Neuroradiol. 2016;22(6):624–37. 27511817 10.1177/1591019916659262 PMC5564353

[15] Jiang LC, Cao JY, Chen M. Coronary artery aneurysm combined with other multiple aneurysms at multiple locations: a case report and systematic review. Medicine (Baltimore). 2017;96(50): e9230. 29390352 10.1097/MD.0000000000009230 PMC5815764

[16] Brown RD Jr, Broderick JP. Unruptured intracranial aneurysms: epidemiology, natural history, management options, and familial screening. Lancet Neurol. 2014;13(4):393–404. 24646873 10.1016/S1474-4422(14)70015-8

[17] Kim T, Lee H, Ahn S, Kwon OK, Bang JS, Hwang G, Kim JE, Kang HS, Son YJ, Cho WS, et al. Incidence and risk factors of intracranial aneurysm: A national cohort study in Korea. Int J Stroke. 2016;11(8):917–27. 27422699 10.1177/1747493016660096

[18] Śliwczyński A, Jewczak M, Dorobek M, Furlepa K, Gołębiak I, Skibińska E, Sarzyńska-Długosz I. An analysis of the incidence and cost of intracranial aneurysm and subarachnoid haemorrhage treatment between 2013 and 2021. Int J Environ Res Public Health. 2023;20(5):3828. 10.3390/ijerph20053828. 36900834 10.3390/ijerph20053828 PMC10001767

[19] Takagi H, Hari Y, Nakashima K, Kuno T, Ando T. Association of aortic and intracranial aneurysm: tweedledum and tweedledee? Eur J Prev Cardiol. 2020;27(19):2272–5. 31698966 10.1177/2047487319886683

[20] Hoh BL, Ko NU, Amin-Hanjani S, Chou S-Y, Cruz-Flores S, Dangayach NS, Derdeyn CP, Du R, Hänggi D, Hetts SW, et al. 2023 Guideline for the management of patients with aneurysmal subarachnoid hemorrhage: a guideline from the american heart association/american stroke association. Stroke. 2023;54(7):e314–70. 37212182 10.1161/STR.0000000000000436

[21] Sakalihasan N, Michel JB, Katsargyris A, Kuivaniemi H, Defraigne JO, Nchimi A, Powell JT, Yoshimura K, Hultgren R. Abdominal aortic aneurysms. Nat Rev Dis Primers. 2018;4(1):34. 30337540 10.1038/s41572-018-0030-7

[22] Arroyo AG, Iruela-Arispe ML. Extracellular matrix, inflammation, and the angiogenic response. Cardiovasc Res. 2010;86(2):226–35. 20154066 10.1093/cvr/cvq049 PMC2856193

[23] Intengan HD, Schiffrin EL. Vascular remodeling in hypertension: roles of apoptosis, inflammation, and fibrosis. Hypertension. 2001;38(3 Pt 2):581–7. 11566935 10.1161/hy09t1.096249

[24] Okamoto E, Couse T, De Leon H, Vinten-Johansen J, Goodman RB, Scott NA, Wilcox JN. Perivascular inflammation after balloon angioplasty of porcine coronary arteries. Circulation. 2001;104(18):2228–35. 11684636 10.1161/hc4301.097195

[25] Chalouhi N, Hoh BL, Hasan D. Review of cerebral aneurysm formation, growth, and rupture. Stroke. 2013;44(12):3613–22. 24130141 10.1161/STROKEAHA.113.002390

[26] Frösen J, Piippo A, Paetau A, Kangasniemi M, Niemelä M, Hernesniemi J, Jääskeläinen J. Remodeling of saccular cerebral artery aneurysm wall is associated with rupture: histological analysis of 24 unruptured and 42 ruptured cases. Stroke. 2004;35(10):2287–93. 15322297 10.1161/01.STR.0000140636.30204.da

[27] Dale MA, Ruhlman MK, Baxter BT. Inflammatory cell phenotypes in AAAs: their role and potential as targets for therapy. Arterioscler Thromb Vasc Biol. 2015;35(8):1746–55. 26044582 10.1161/ATVBAHA.115.305269 PMC4514552

[28] Lu H, Rateri DL, Bruemmer D, Cassis LA, Daugherty A. Novel mechanisms of abdominal aortic aneurysms. Curr Atheroscler Rep. 2012;14(5):402–12. 22833280 10.1007/s11883-012-0271-y PMC3436976

[29] Raffort J, Lareyre F, Clément M, Hassen-Khodja R, Chinetti G, Mallat Z. Monocytes and macrophages in abdominal aortic aneurysm. Nat Rev Cardiol. 2017;14(8):457–71. 28406184 10.1038/nrcardio.2017.52

[30] Engin AB, Nikitovic D, Neagu M, Henrich-Noack P, Docea AO, Shtilman MI, Golokhvast K, Tsatsakis AM. Mechanistic understanding of nanoparticles’ interactions with extracellular matrix: the cell and immune system. Part Fibre Toxicol. 2017;14(1):22. 28646905 10.1186/s12989-017-0199-z PMC5483305

[31] Arenas Gómez CM, Sabin KZ, Echeverri K. Wound healing across the animal kingdom: Crosstalk between the immune system and the extracellular matrix. Dev Dyn. 2020;249(7):834–46. 32314465 10.1002/dvdy.178 PMC7383677

[32] Sutherland TE, Dyer DP, Allen JE. The extracellular matrix and the immune system: a mutually dependent relationship. Science. 2023. 10.1126/science.abp8964. 36795835 10.1126/science.abp8964

[33] Gullberg D, Velling T, Sjöberg G, Sejersen T. Up-regulation of a novel integrin alpha-chain (alpha mt) on human fetal myotubes. Dev Dyn. 1995;204(1):57–65. 8563026 10.1002/aja.1002040108

[34] Tiger CF, Fougerousse F, Grundström G, Velling T, Gullberg D. alpha11beta1 integrin is a receptor for interstitial collagens involved in cell migration and collagen reorganization on mesenchymal nonmuscle cells. Dev Biol. 2001;237(1):116–29. 11518510 10.1006/dbio.2001.0363

[35] Zhu CQ, Popova SN, Brown ER, Barsyte-Lovejoy D, Navab R, Shih W, Li M, Lu M, Jurisica I, Penn LZ, et al. Integrin alpha 11 regulates IGF2 expression in fibroblasts to enhance tumorigenicity of human non-small-cell lung cancer cells. Proc Natl Acad Sci U S A. 2007;104(28):11754–9. 17600088 10.1073/pnas.0703040104 PMC1913903

[36] Bieber K, Bezdek S, Gupta Y, Vorobyev A, Sezin T, Gross N, Prüssmann J, Sayegh JP, Becker M, Mousavi S, et al. Forward genetics and functional analysis highlight Itga11 as a modulator of murine psoriasiform dermatitis. J Pathol. 2023;261(2):184–97. 37565309 10.1002/path.6162

